# Catarrhal Conjunctivitis

**Published:** 1847-01

**Authors:** 


*Catarrhal Conjunctivitis.*—This man, a coal-heaver by occupation, presents himself for a difficulty which is at once easily recognized as conjunctivitis, or an inflammation of one of the subdivisions of the tegumentary system, viz., the mucous tissue, which in this particular locality receives the name of conjunctiva. The extent and relations of this membrane you are familiar with; it invests the anterior surface of the eyeball, is reflected over the inner surface of the eyelids, passes into the Meibomian follicles, and is, through the puncta lachrymalia, and ductus lachrymalis, continuous with the great gastro-pulmonary mucous membrane. Now this conjunctiva, like all other mucous membranes, is liable to be attacked with common inflammation, superinduced by atmospheric changes, or sudden alterations of heat or cold, excessive use of the eyes, the introduction of foreign bodies, etc. etc. Authors make several varieties of this affection, such as the ordinary simple catarrhal conjunctivitis, (an example of which you have before you,) the scrofulous, the gonorrhœal, the granular, etc. Now as to the symptoms : they are first, the ordinary symptoms of inflammation, pain, redness, heat and swelling. The special symptoms are, diminution of the palpebral fissure, intolerance of light, (this symptom is most marked in the scrofulous form,) increased lachrymation, and an increase in the quantity of the mucous secretion, which is sometimes puriform. The Meibomian secretion becomes altered in quantity and quality, becomes concrete, and is entangled by the eyelashes. During the day, it is washed away by the other secretions, but at night concretes on the margins of the palpebræ, causing them to adhere, and in awaking, it is with difficulty the patient is enabled to open his eyes. This affection, when uncomplicated, has received the name of *Lippitudo*. The increased vascularity is an important symptom in conjunctivitis; the vessels are arranged in the form of a net work, and are of a scarlet color, and by moving the conjunctiva, these tortuous vessels move also. This single symptom is important in distinguishing this affection from sclerotitis, in which the vessels are of a pale color, not reticulated, but arranged in radii diverging from the margin of the cornea, and do not follow the motions of the conjunctiva, but remain stationary. Inflammation may be communicated by contiguous sympathy from the conjunctiva to the sclerotica, and vice versa. The pain in simple conjunctivitis is less acute than in sclerotitis. As to the diagnosis, I have already said enough for the present on that point; the iris is healthy, not changed in color, and the pupil retains its natural form; these symptoms, with the absence of circum-orbital pain, would prevent you from confounding it with iritis. As to the prognosis; if the case is properly treated, it is good; if neglected, it is bad, and why? It will run into the granular form, which is exceedingly, I say exceedingly obstinate in yielding to treatment; and as a result of this granular condition, you will have opacity of the cornea, a very unpleasant termination. As to the treatment, the simpler is the better; keep the patient quiet, and the eyes at rest, direct a low, unstimulating diet, with a moderately stimulating collyrium of the Sulphas Zinci. This will often of itself succeed, or it may become necessary to resort to more energetic antiphlogistic means, such as blood-letting, local or general, active purgation, counter irritation, etc. In this case I shall simply recommend rest, and a powder of ℞ Protochlor. Hydrarg: Pulv. Doveris. aa. gr. v. M., to be taken at night. Topically, I would freely employ cold or warm water, whichever is most grateful, and the collyrium before alluded to, of Sulphate or Acetate or Zinc, and to correct the vitiated condition of the Meibomian follicles, I would anoint the margins of the eyelids at bed-time with dilute citrine ointment, or the Ung. Hyd. Peroxidi, in the proportion of 20 grains to the ounce. There is one remark with regard to collyria, and that is that the smarting they occasion, should cease in ten minutes; if it continue longer, your application will do harm; it is too strong and must be diluted.

